# Supplementary figures and images for: Metabolomic analyses uncover an inhibitory effect of niclosamide on mitochondrial membrane potential in cholangiocarcinoma cells
Source: PeerJ. 2023 Nov 22;11:e16512. doi: 10.7717/peerj.16512 (PMC10676079; doi:10.7717/peerj.16512)

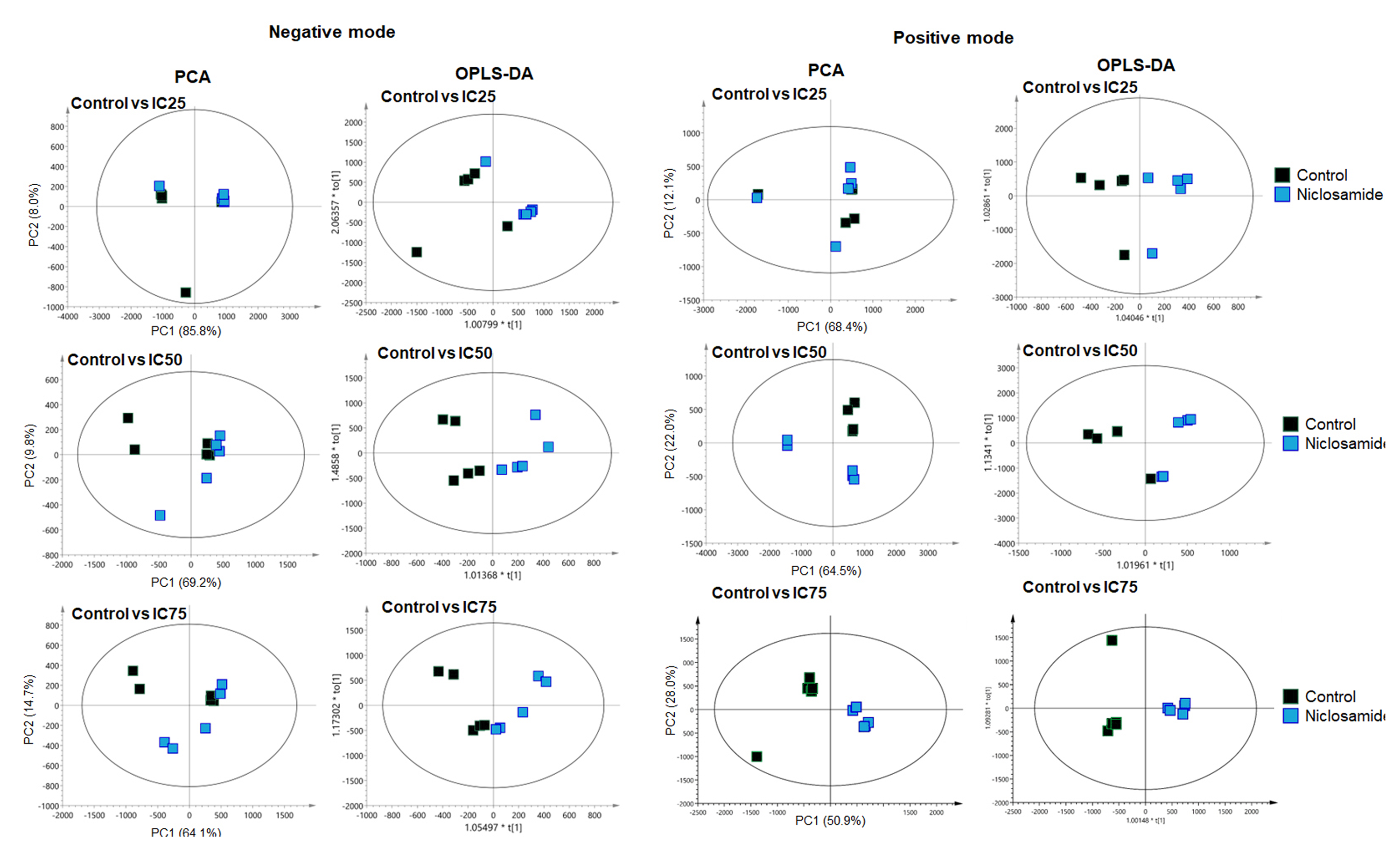

Supplement: Supplemental Information 1 — The OPLS-DA score plot of niclosamide-treated at IC25, IC50 and IC75 in negative mode showed R2X = 0.936, Q2 = 0.204, R2X = 0.772, Q2 = 0.409 and R2X = 0.774, Q2 = 0.328, respectively. The OPLS-DA score plot of niclosamide-treated at IC25, IC50 and IC75 in positive mode showed R2X = 0.556, Q2 = 0.15, R2X = 0.534, Q2 = 0.43 and R2X = 0.749, Q2 = 0.925, respectively. [file peerj-11-16512-s001.png]

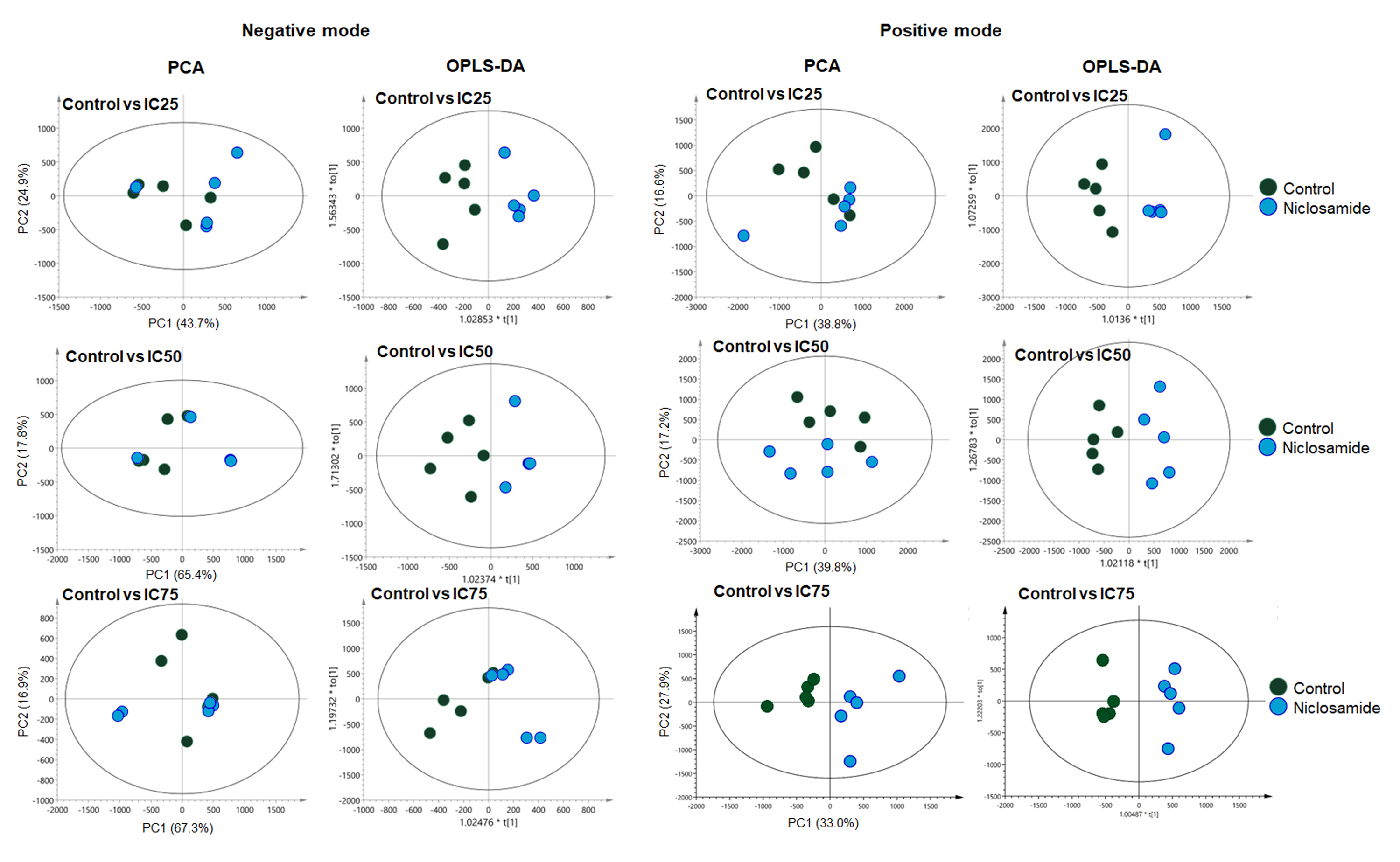

Supplement: Supplemental Information 2 — The OPLS-DA score plot of niclosamide-treated at IC25, IC50 and IC75 in negative mode showed R2X = 0.673, Q2 = −0.137, R2X = 0.878, Q2 = −0.179 and R2X = 0.923, Q2 = −0.595, respectively. The OPLS-DA score plot of niclosamide-treated at IC25, IC50 and IC75 in positive mode showed R2X = 0.53, Q2 = 0.76, R2X = 0.537, Q2 = 0.74 and R2X = 0.393, Q2 = 0.958, respectively. [file peerj-11-16512-s002.png]

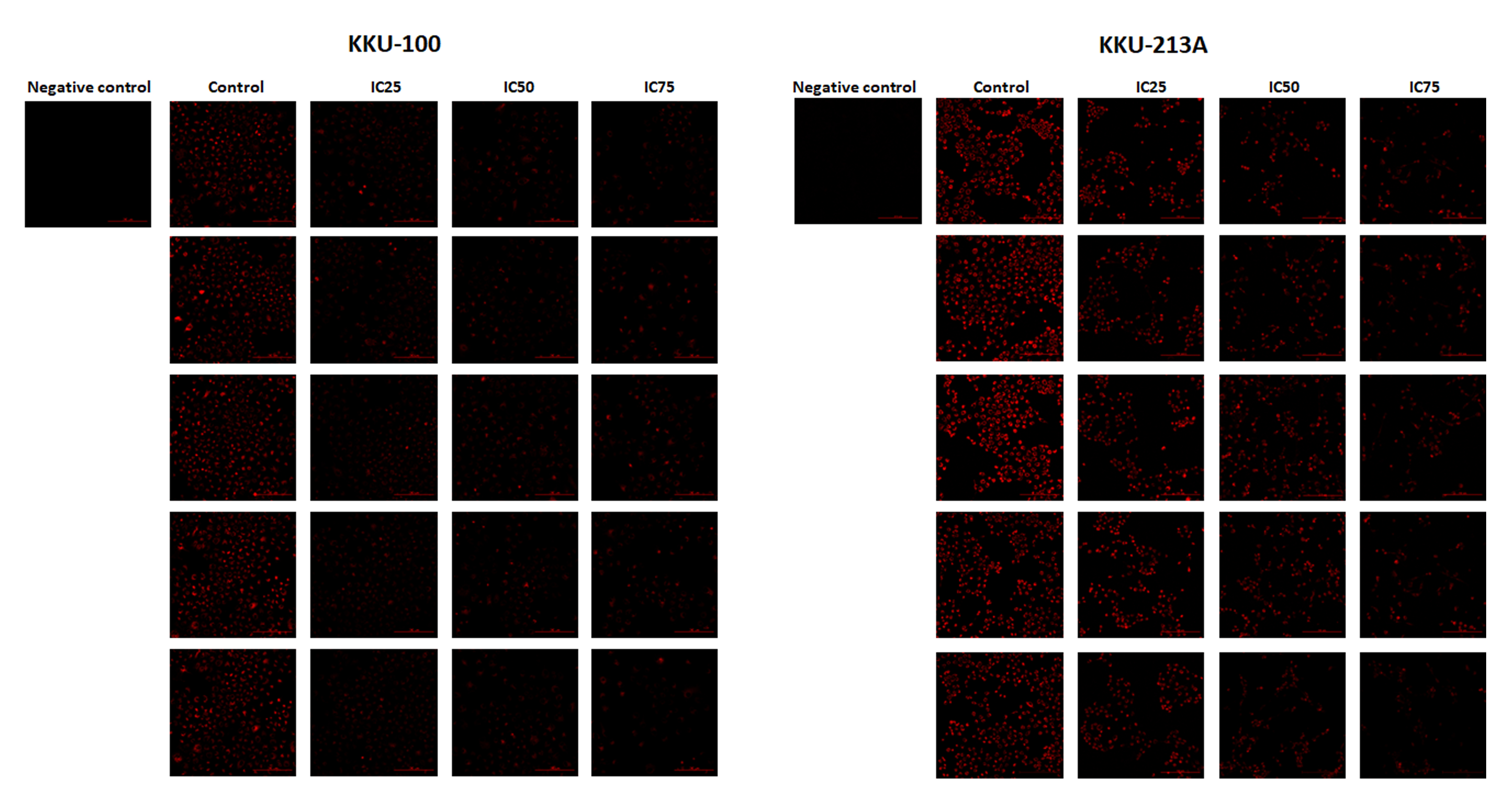

Supplement: Supplemental Information 4 [file peerj-11-16512-s004.jpg]
